# Photoelectrochemical Biosensor Based on 1D In_2_O_3_ Tube Decorated with 2D ZnIn_2_S_4_ Nanosheets for Sensitive PSA Detection

**DOI:** 10.3390/nano15110855

**Published:** 2025-06-03

**Authors:** Huihui Shi, Jianjian Xu, Yanhu Wang

**Affiliations:** 1 Key Laboratory of MEMS of Ministry of Education, Southeast University, Nanjing 210096, China; shihuihui@seu.edu.cn; 2 Shandong Analysis and Test Center, Qilu University of Technology (Shandong Academy of Sciences), Jinan 250014, China; 3 Department of Food and Drug, Weihai Ocean Vocational College, Weihai 264300, China; jjjcs@whovc.edu.cn

**Keywords:** photoelectrochemical, In_2_O_3_-ZnIn_2_S_4_, heterointerfaces, charge separation, PSA

## Abstract

In photoelectrochemical (PEC) biosensing, efficient electron-hole separation is crucial to obtain preferred photocurrent response and analytical performance; thus, constructing developed heterointerfaces with high carrier transfer efficiency is an effective method for sensitive evaluation of analytes. Herein, a 1D ZnIn_2_S_4_ nanosheet-decorated 2D In_2_O_3_ tube was developed to integrate with a prostate antigen (PSA)-sensitive aptamer for sensitive PSA antigen detection. 1D In_2_O_3_ tubes were first prepared by two-step hydrothermal and annealing methods, followed by the in-situ growth of ZnIn_2_S_4_ nanosheets. Morphology, optical properties, structure, and PEC performance of prepared In_2_O_3_-ZnIn_2_S_4_ were characterized by scanning electron microscopy, transmission electron microscopy, ultraviolet–visible spectrophotometry, X-ray diffraction, X-ray photoelectron spectroscopy, and an electrochemical workstation. Benefiting from the photoelectric effect and specific 1D/2D hierarchical structure, In_2_O_3_-ZnIn_2_S_4_ displayed enhanced optical absorption and photocarrier separation, thus a superior photoelectrochemical response. Proposed bioassay protocol possessed a linear range from 0.001 to 50 ng/mL and a detection limit at 0.00037 ng/mL. In addition, this biosensor exhibited satisfactory anti-interface ability and stability, which also could be extended to other quantitative platforms for detecting other proteins.

## 1. Introduction

Photoelectrochemical (PEC) bioanalysis, featuring high sensitivity and simple equipment, has been used to quantify various analytes through photocurrent variation [1,2]. Essentially, sensing photocurrent signals strongly depends on the light–electricity conversion process and carrier separation efficiency of photoactive materials. Thus, besides recognition units, photoactive materials with superior PEC performance are crucial factors when designing sensitive PEC biosensors. Indium oxide (In_2_O_3_), as a typical moderate-band-gap n-type semiconductor, has been widely used in photoelectrochemistry due to its high chemical stability, low resistivity, low toxicity, and easy preparation [3,4]. However, pure In_2_O_3_ has always suffered from poor PEC performance, which mainly contributed to high carrier recombination rate, limited photon utilization, and deficient surface active sites. Multiple reports have demonstrated heterojunction construction by coupling other semiconductors with different band gaps, which could prominently promote the photogenerated electrons and hole migration at the heterojunction interface, thus enhancing the PEC performance of In_2_O_3_-based materials [5,6,7,8].

Up to now, multiple In_2_O_3_-based heterostructures, such as In_2_O_3_/Co_3_O_4_ [6], In_2_O_3_/g-C_3_N_4_ [9], In_2_O_3_/CdS [10], and In_2_O_3_/In_2_S_3_ [11], have been prepared with improved photoelectric transformation efficiency. However, mismatched lattice between these semiconductors and In_2_O_3_ usually induced heterointerface impedance and restricted carrier separation. Compared with the above mentioned semiconductors, 2D layered ZnIn_2_S_4_ has drawn increasing attention due to high specific surface areas, short carrier migration pathways, rich active sites, and similar lattice parameters [12,13]. The high lattice matching degree of In_2_O_3_ and ZnIn_2_S_4_ is conducive to the formation of a compact interface and thus greatly decreases the charge immigration impedance of the heterointerface, and finally, facilitates spatial charge separation [14]. A significant challenge is that ZnIn_2_S_4_ sheets tend to agglomerate into nanoclusters, causing a low specific surface area and reduced active sites [15,16]. Supporting carriers, including CdS nanocube, NiMoOx nanorod, FeWO_4_ flower, and Co_9_S_8_ tube, favor lamellar growth and inhibit agglomerates [17,18,19,20]. Thus, it is of great favor to employ a 1D In_2_O_3_ tube as the supporter to offer ana abundant area for nanosheet growth and conduction paths for photogenerated carrier transport. With such a design, branched ZnIn_2_S_4_ nanosheets on In_2_O_3_ tubes could facilitate solar-light harvesting, and an intimate heterointerface could modulate the migration pathway for extending the photogenerated charge lifetime.

Prostate antigen (PSA) level, as an early portent of prostate dysfunction, is associated with prostate cancer, underscoring the importance of sensitive PSA detection (<4 ng/mL—traditionally considered normal). Multiple sensing strategies have been developed for PSA quantification. For example, a sandwich-type electrochemical immunosensor with delaminated MXene@AuNPs as signal amplification was reported for PSA ultra-sensitive analysis [21]. Another peptide–antibody sandwich electrochemical biosensor based on the MnO_2_-functionalized COF was successfully constructed [22]. A meta-nano-channel silicon field-effect biosensor fabricated on silicon-on-insulator wafers was proposed for label-free PSA sensing [23]. These studies achieved ultra-sensitive PSA detection, which is usually accompanied by complex sensor modification processes and cumbersome sensing procedures.

**Figure 1:**
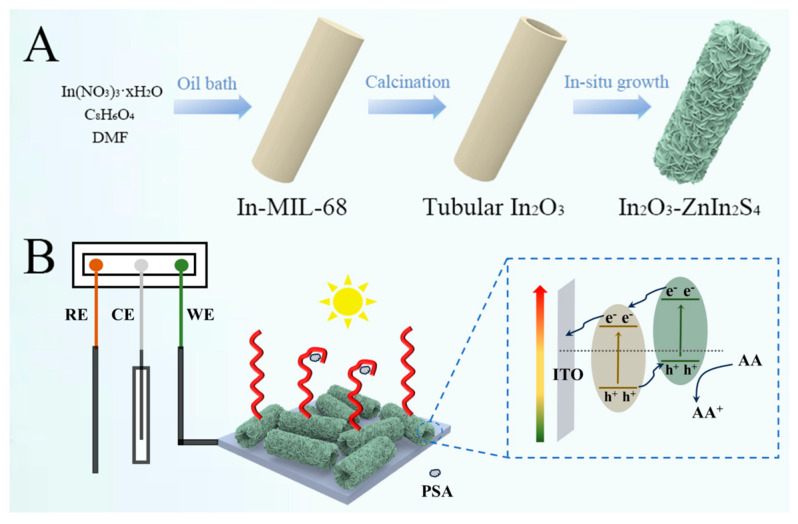
(**A**) Schematic illustration of In_2_O_3_-ZnIn_2_S_4_ synthesis route. (**B**) Schematic illustration of PEC electrode and sensing mechanism.

In this work, a 1D/2D heterostructure was designed by decorating ZnIn_2_S_4_ nanosheets onto In_2_O_3_ tubes to integrate with the PSA-sensitive aptamer for PSA antigen quantification. The overall heterostructure preparation process involved a template method and in-situ growth strategy where MIL-68 (Materials of Institute Lavoisier MOF materials) was transformed into tubular In_2_O_3_ and further modified with ZnIn_2_S_4_ nanosheets (Figure 1A). Under visible light, nanosized heterogeneous interface favored the effective separation and migration of electron-hole pairs, endowing the hybrid with desirable PEC performance (Figure 1B). During this process, the photogenerated electrons accumulated in the conduction band of In_2_O_3_ while photogenerated holes accumulated in the valence band of ZnIn_2_S_4_. Efficient separation of electrons and holes was achieved with ascorbic acid as the electron donor to scavenge holes. Furthermore, a modified aptamer on the PEC electrodes could specifically bind with the target; thus, a relevance between PSA concentration and photocurrent response was established. Finally, the PEC sensing platform displayed a linear response range from 0.001 to 50 ng/mL and a detection limit at 0.00037 ng/mL. This platform also possessed satisfying selectivity and stability. Such a simple construction process and efficient sensing protocol have great potential in other biomolecule quantifications.

## 2. Materials and Methods

### 2.1. Reagents and Apparatus

All reagents are of analytical reagent grade and are directly used for all experiments. Ultrapure water (resistivity ≥ 18.25 MΩ cm) was obtained from a Lichun water purification system. Glass with a 150 nm FTO layer (~15 Ω/square resistance) was purchased from Xiamen FTO Photoelectricity Industry (Xiamen, China). Nitrate hydrate, N. N-dimethylformamide, chitosan, bovine serum albumin (BSA), and glutaraldehyde were offered by Sinopharm Chemical Reagent Co., Ltd. (Shanghai, China); 1,4-benzenedicarboxylic acid, zinc chloride, indium chloride, and thioacetamide were bought from Macklin Reagent Co., Ltd. (Shanghai, China); 0.01 M PBS (pH = 7.4) buffer was employed as incubation buffer.

PSA aptamer: (5′-NH2-(CH2)6-ATTAAAGCTCGCCATCAAATAGC-3′)

Scanning electron microscopy (SEM) and transmission electron microscopy (TEM) images were obtained using the QUANTA FEG 250 thermal field emission scanning electron microscopy (FEI Co., Hillsboro, OR, USA) and Hitachi H600 with 200 kV acceleration voltage. Elemental mapping images were recorded using an EDX spectroscope attached to a TEM. X-ray diffraction (XRD) patterns and X-ray photoelectron spectroscopy (XPS) spectra were collected by a D8 advance diffractometer system equipped with Cu Ka radiation (Bruker Co., Bremen, Germany) and an ESCALAB 250Xi photoelectron spectrometer, respectively. UV-vis absorption measurements were achieved by a UH-4150 ultraviolet–visible spectrophotometer (Hitachi, Tokyo, Japan). And the photocurrent and electrochemical impedance spectroscopy (EIS) were measured on a CHI 660D electrochemical workstation (Shanghai Chenhua Instruments Corporation, Shanghai, China) with a three-electrode system. Photocurrents were recorded under a 500 W xenon lamp (100 mW·cm^−2^) illumination.

### 2.2. Preparation of Hollow Tubular In_2_O_3_

Tubular In_2_O_3_ was synthesized by sequential hydrothermal and thermal methods. Initially, indium nitrate hydrate (0.06 g) and 1,4-benzenedicarboxylic acid (0.06 g) were dissolved in 40 mL N,N-dimethylformamide, and further stirred for 5 min at room temperature. The resultant solution was heated at 120 °C for 30 min, followed by filtration and washed with ethanol to obtain white MIL-68. Finally, an annealing procedure at 500 °C was performed for 2 h in a muffle furnace, yielding light yellow In_2_O_3_ tubes.

### 2.3. Synthesis of Branched-Sheet Embedded Tubular In_2_O_3_-ZnIn_2_S_4_

Briefly, as-prepared In_2_O_3_ tubes (0.1 g) and ZnIn_2_S_4_ precursor (0.05 g of zinc chloride, 0.23 g indium chloride, and 0.24 g thioacetamide) were fully dissolved in 30 mL deionized water and then stirred continuously for 30 min at 80 °C in an oil bath. The obtained precipitate In_2_O_3_-ZnIn_2_S_4_ was collected, centrifugated, washed with deionized water, and dried under vacuum. Nanosheet-based ZnIn_2_S_4_ clusters were synthesized using the same method without the addition of In_2_O_3_.

### 2.4. Fabrication of Sensing Platform and Analysis Protocol

FTO glass was pre-cleaned sequentially with acetone, ethanol, and deionized water under violent ultrasonication before use. In order to obtain an attractive PEC signal, 1 mL of prepared In_2_O_3_-ZnIn_2_S_4_ solution was spin-coated onto the FTO glass, followed by drying under an infrared lamp for 30 min. A volume of 50 μL of chitosan aqueous solution (0.08 wt.%) in 1% acetic acid was dropped onto the FTO electrode, and then, 5 wt.% glutaraldehyde solution was applied onto the electrode to trigger amino groups for subsequent biomolecule modification. After that, the electrode was incubated with 20 µL of 1 µM aptamer for 70 min at 4 °C by adding the adapter solution onto the electrode surface, followed by the addition of 10 μL of 2 wt.% BSA to block non-specific binding sites. During the 70-min adapter incubation process, the adapter was connected to the electrode via glutaraldehyde-mediated Schiff base. The obtained working electrode was stored at 4 °C and denoted as FTO/In_2_O_3_-ZnIn_2_S_4_/aptamer/BSA. Before PEC measurements, the FTO/In_2_O_3_-ZnIn_2_S_4_/aptamer/BSA was incubated with 20 μL PSA at room temperature for 30 min. Notably, FTO electrodes were thoroughly cleaned with PBS buffer (pH = 7.4, 0.01 M) after each step. All PEC signals were generated by a typical three-electrode system (FTO working electrode, counter electrode, and reference electrode) in 0.1 M ascorbic acid (AA) solution.

### 2.5. Detection Limit Calculation

The detection limit was obtained by the formula I_LOD_ = I_blank_ + 3S_blank_, where I_blank_ and S_blank_ are the average photocurrent of 10 independent samples (without PSA) and the corresponding standard deviation, respectively. Then, I_LOD_ was brought into the regression curve to obtain the detection limit.

## 3. Results and Discussion

### 3.1. Morphology and Structure Characterization

**Figure 2:**
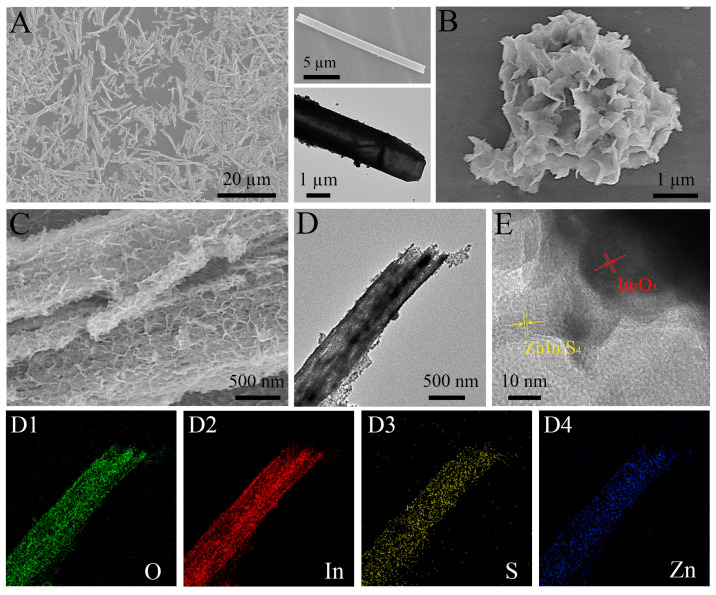
SEM images of (**A**) pure In_2_O_3_, (**B**) pure ZnIn_2_S_4_, and (**C**) hybrid In_2_O_3_-ZnIn_2_S_4_. (**D**) TEM image of In_2_O_3_-ZnIn_2_S_4_ and elemental mappings of O, In, S, and Zn elements (**D1**–**D4**). (**E**) HRTEM image of In_2_O_3_-ZnIn_2_S_4_.

Hybrid In_2_O_3_-ZnIn_2_S_4_ was synthesized by the template method and in-situ growth technique and further investigated by scanning electron microscope (SEM), transmission electron microscope (TEM), and high-resolution TEM (HRTEM). Apparently, a highly dispersed tube microstructure with well-defined tube walls and cavities was found for In_2_O_3_ (Figure 2A), providing adequate space for ZnIn_2_S_4_ growth. While pure ZnIn_2_S_4_ displayed irregular clusters assembled by a large number of nanosheets (Figure 2B and Figure S1). After loading the nanosheets onto In_2_O_3_, the as-prepared composite exhibited uniform and dense coverage of ultrathin nanosheets, and O, In, S, and Zn elements were evenly distributed on the single tube (Figure 2C,D,(D1–D4)). Significantly, compared to self-assembled ZnIn_2_S_4_ nanosheet clusters, interconnected nanosheets are beneficial for increasing specific surface area and providing sufficient active sites. Furthermore, a tight interfacial junction between In_2_O_3_ and ZnIn_2_S_4_ was successfully constructed where lattice spacing of 0.29 and 0.32 nm were ascribed to the In_2_O_3_ (2 2 2) and ZnIn_2_S_4_ (1 0 2) crystal planes, respectively (Figure 2E) [24]. These images demonstrated the successful fabrication of a branched sheet embedded tubular hybrid and heterostructure.

**Figure 3:**
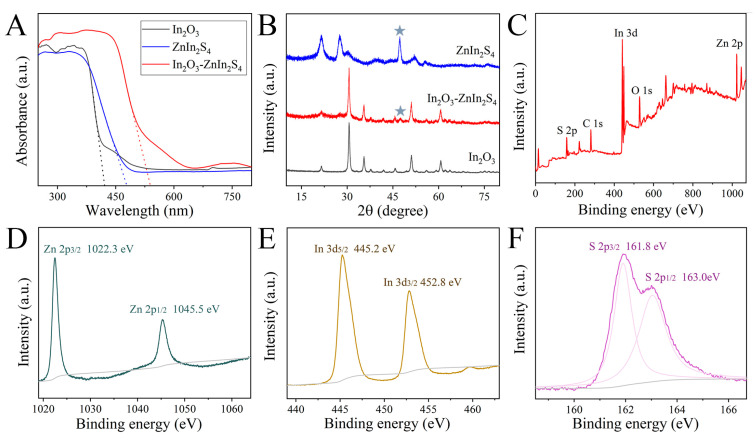
(**A**) UV-vis absorption spectra and (**B**) XRD patterns of In_2_O_3_, ZnIn_2_S_4_, and In_2_O_3_-ZnIn_2_S_4_. Grey stars in (**B**) represent one of the characteristic peaks of ZnIn_2_S_4_. High-resolution XPS spectra of (**C**) In_2_O_3_-ZnIn_2_S_4_, (**D**) Zn 2p, (**E**) In 3d, and (**F**) S 2p.

Optical properties, crystalline phases, and chemical states were also measured by UV-vis absorption spectra, X-ray diffraction (XRD), and X-ray photoelectron spectroscopy (XPS), respectively. As shown in Figure 3A, a typical absorption edge at ~425 nm and poor light-harvesting capacity in the visible light region were demonstrated for pure In_2_O_3_. For hybrid In_2_O_3_-ZnIn_2_S_4_, a robust photo-absorption in the UV and visible light region to ~542 nm was obtained. Using the Tauc plot method [25], the bandgap energies (Eg) of In_2_O_3_, ZnIn_2_S_4_, In_2_O_3_-ZnIn_2_S_4_ were calculated to be 2.9, 2.58, and 2.29 eV, respectively. Furthermore, XRD patterns of the above-mentioned materials are shown in Figure 3B. Nine distinct diffraction peaks at 21.5°, 30.6°, 35.5°, 37.7°, 41.8°, 43.7°, 51.1°, 55.9°, and 60.7° in black curve were well-matched with characteristic standard In_2_O_3_ data (JCPDS No. 06-0416) [14]. Except for diffraction peaks of In_2_O_3_, an additional peak at 47.2° gathered from In_2_O_3_-ZnIn_2_S_4_ was assigned to the (1 1 0) crystal plane of pure ZnIn_2_S_4_. To probe the elemental valence details, XPS was performed with the C 1s peak as the standard reference. In the survey spectrum, the presence of In, O, Zn, and S elements was confirmed, consistent with the above-mentioned mapping diagram. Specifically, two binding energies at 1022.3 and 1045.5 eV were attributed to the Zn 2p3/2 and Zn 2p1/2 of Zn^2+^ chemical states. Two prominent peaks presented at 445.2 eV (In 3d5/2) and 452.8 eV (In 3d3/2) were recorded, which were similar to that of standard In^3+^. The S 2p spectrum could be deconvoluted into two characteristic S^2−^ peaks at 161.8 and 163.0 eV. In a word, the above-mentioned results matched each other, illustrating the successful fabrication of tubular In_2_O_3_-ZnIn_2_S_4_.

### 3.2. PEC and EIS Behaviors

**Figure 4:**
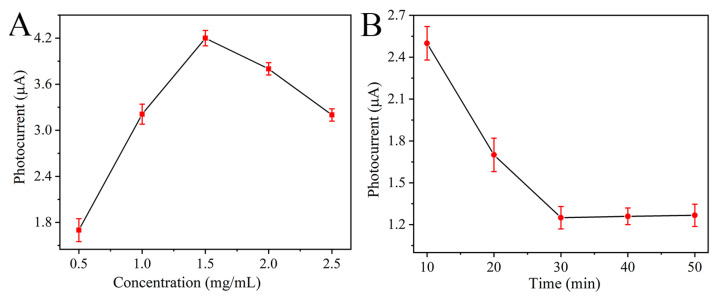
(**A**) Photocurrent responses of FTO/In_2_O_3_-ZnIn_2_S_4_ (0.5, 1, 1.5, 2, 2.5 mg/mL) in 0.1 M ascorbic acid solution. (**B**) Photocurrent responses of FTO/In_2_O_3_-ZnIn_2_S_4_/aptamer/BSA after 1 ng/mL PSA incubation (10, 20, 30, 40, 50 min) in 0.1 M ascorbic acid solution.

To obtain a desirable initial signal for subsequent PEC biosensing, a 1D ZnIn_2_S_4_ nanosheet-decorated 2D In_2_O_3_ tube served as the photosensitive material. The interconnected nanosheets grown in situ on the tube exhibit a high specific surface area and provide more carrier migration pathways, thus endowing the photoactive material with a high photocurrent. The entire sensing platform was constructed after modifying the PSA-sensitive aptamer for PSA-sensitive detection. During the platform construction and sensing process, two key factors, including the In_2_O_3_-ZnIn_2_S_4_ concentration and PSA antigen incubation time, were investigated, and the results are described in Figure 4. With the increase in In_2_O_3_-ZnIn_2_S_4_ concentration, the photocurrent value displayed an inverted V-shaped pattern, and the maximal signal was obtained at 1.5 mg mL^−1^ In_2_O_3_-ZnIn_2_S_4_. Additionally, Figure 4B exhibited the effect of PSA incubation time on PEC response. The photocurrent response decreased continuously from 10 to 50 min, and 30 min was chosen as the incubation time.

**Figure 5:**
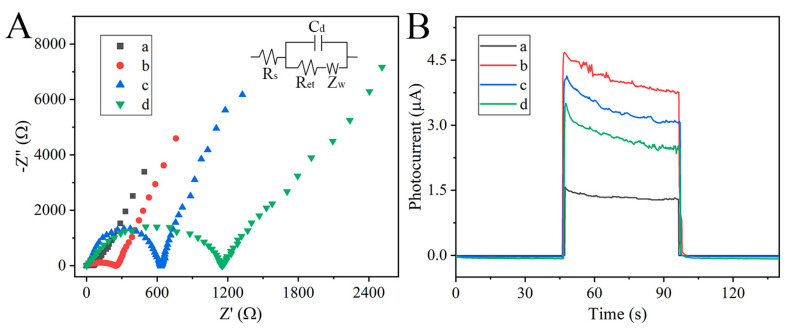
(**A**) EIS spectra of (a) FTO, (b) FTO/In_2_O_3_-ZnIn_2_S_4_, (c) FTO/In_2_O_3_-ZnIn_2_S_4_/aptamer, and (d) FTO/In_2_O_3_-ZnIn_2_S_4_/aptamer/BSA in 5.0 mM [Fe(CN)_6_]^3−^/^4−^ containing 0.1 M potassium chloride. (**B**) Photocurrent responses of (a) FTO/In_2_O_3_, (b) FTO/In_2_O_3_-ZnIn_2_S_4_, (c) FTO/In_2_O_3_-ZnIn_2_S_4_/aptamer, and (d) FTO/In_2_O_3_-ZnIn_2_S_4_/aptamer/BSA in 0.1 M ascorbic acid solution.

The stepwise construction process of the PSA-sensing platform was estimated by electrochemical impedance spectroscopy (EIS,) where the larger radius always means a lower charge transfer rate (Figure 5A). The corresponding equivalent circuit, including solution resistance (R_s_), double-layer capacitance (C_d_), the electrode transfer resistance (R_et_), and the Warburg impedance (Z_W_) (Table S1), is shown as an inset. Warburg impedance reflects charge diffusion from solution to electrode interface. During successive modification processes, the Warburg impedance gradually increased, indicating a continuous decrease in diffusion rate. R_et_ is a critical signal revealing the interfacial properties of the modified procedure. It could be observed that the electron-transfer resistance (Ret) elevated dramatically after immobilization of the In_2_O_3_-ZnIn_2_S_4_ complex onto the FTO surface due to their low conductivity. With the identify element and blocking agent modification progress, Ret showed an upward trend. This is because the large steric hindrance of the aptamer and BSA diminished the charge transfer capacity. Moreover, the transient photocurrent responses were also measured using 0.1 M ascorbic acid solution as the sacrificial agent (Figure 5B). As expected, FTO/In_2_O_3_-ZnIn_2_S_4_ (red curve, ~4.13 µA) showed significant photocurrent enhancement compared with FTO/In_2_O_3_ (black curve, ~1.33 µA) thanks to the heterointerfaces promoting photogenerated electron-hole separation. Under visible light radiation, photoinduced electrons and holes were generated on the In_2_O_3_ and ZnIn_2_S_4_ surfaces. Driven by the internal electric field, photoinduced electrons and holes accumulated on the conduction band of ZnIn_2_S_4_ and valence band of In_2_O_3_, respectively. An enhanced anodic photocurrent was obtained, and holes were captured by an electron donor (ascorbic acid). After the photoelectrode was incubated with a non-conductive aptamer and BSA, the continuously decreasing photocurrent value was obtained (blue curve, ~3.41 µA; green curve, ~2.76 µA). The EIS and PEC response demonstrated continuous fixation of biomolecules on the FTO electrode.

### 3.3. Analytical Performance

**Figure 6:**
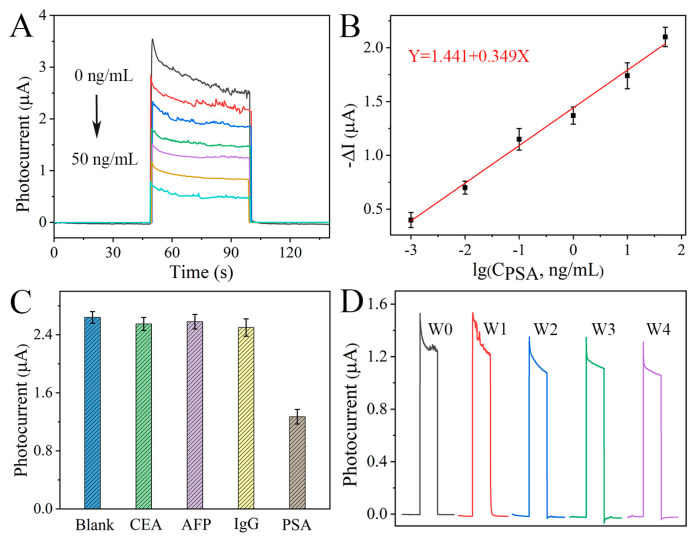
(**A**) Photocurrent responses of FTO/In_2_O_3_-ZnIn_2_S_4_/aptamer/BSA at different PSA concentrations (0, 0.001, 0.01, 0.1, 1, 10, 50 ng/mL) and (**B**) the calibration curve between -ΔI and logarithm of PSA concentration. (**C**) Photocurrent response of proposed biosensing platform in the presence of 1 ng/mL CEA, AFP, IgG, and blank. (**D**) Photocurrent of PEC biosensor at different storage times from 0 to 4 weeks (W0–W4).

Based on this well-designed PEC biosensor, we further explored its capacity for the quantification of PSA antigen. According to the analysis protocol mentioned in the experimental section, PSA at different concentrations was applied to photoelectrodes and acquired PEC signals were analyzed. After incubation with 0.001 ng/mL PSA, the photocurrent intensity abated (red curve in Figure 6A). This may be because aptamer–PSA binding events enlarged steric hindrance and inhibited the diffusion of ascorbic acid to the electrode surface. As the PSA concentration increased from 0.001 to 50 ng/mL, the photocurrent intensity gradually decreased. In other words, there was an excellent negative correlation between photocurrent and logarithmic value of PSA concentrations, and the corresponding regression curve was −ΔI = 1.441 + 0.349lgC_PSA_ (ng/mL) (Figure 6B). This proposed PEC sensing platform possessed a linearity (R^2^) at 0.991 with a detection limit at 0.00037 ng/mL (S/N = 3). Such performance is primarily because of rapid photoinduced charge separation and specific biometric events between the aptamer and the PSA antigen.

To further assess proposed PEC biosensors, both selectivity and stability were monitored. As illustrated in Figure 6C, significant photocurrent change only appeared in the presence of 1 ng/mL PSA, not other 1 ng/mL substances, including carcinoembryonic antigen (CEA), alpha fetoprotein (AFP), and immunoglobulin G (IgG). This result indicates that those interferences had almost no impact on the sensing performance. Moreover, the photocurrent response of the FTO/In_2_O_3_-ZnIn_2_S_4_/aptamer/BSA electrodes at the 4th week maintained 85% of its original value (Figure 6D). Results indicate acceptable storage stability.

## 4. Conclusions

In summary, we successfully constructed an effective PEC biosensor based on FTO/In_2_O_3_-ZnIn_2_S_4_ sensitization structure and a PSA-sensitive aptamer for sensitive analysis of PSA. In_2_O_3_ tubes were first prepared by a two-step hydrothermal and annealing method, followed by the in-situ growth of ZnIn_2_S_4_ nanosheets. In this process, the generated tubular composite established efficient energy-level matching between In_2_O_3_ and ZnIn_2_S_4_. These intimate interface contacts inhibited the photocarrier recombination and made effective migration of photoinduced electrons and holes, thus obtaining desirable initial PEC signals. Additionally, biological binding sites on the hybrid In_2_O_3_-ZnIn_2_S_4_ contributed to the aptamer–protein event and further achieved PSA sensing. The constructed PEC biosensor presented a wide detection range from 0.001 to 50 ng/mL, with a detection limit down to 0.00037 ng/mL. The developed PEC sensing platform has high sensitivity, satisfying selectivity and stability, and guides the optimal PEC electrode construction for PSA detection.

## Data Availability

Data will be made available upon request from the corresponding author.

## Supplementary Materials

The following supporting information can be downloaded at: https://www.mdpi.com/article/10.3390/nano15110855/s1, Experimental section (reagents and apparatus); Figure S1: Enlarged SEM image of ZnIn2S4. Table S1: Key data of EIS measurement in this study. In this model, electron-transfer resistance is the main factor, thus the solution resistance value is artificially zeroed to better demonstrate the change of electron-transfer resistance.

## References

[1] Homer M.K., Kuo D.Y., Dou F.Y., Cossairt B.M. (2022). Photoinduced charge transfer from quantum dots measured by cyclic voltammetry. J. Am. Chem. Soc..

[2] Qin Y., Zhang J., Tan R., Wu Z., Liu M., Li J., Xu M., Gu W., Zhu C., Hu L. (2023). Small-molecule probe-induced in situ-sensitized photoelectrochemical biosensor for monitoring α-Glucosidase activity. ACS Sens..

[3] Ding H., Feng Y., Xu Y., Xue X., Feng R., Yan T., Yan L., Wei Q. (2022). Self-powered photoelectrochemical aptasensor based on MIL-68(In) derived In_2_O_3_ hollow nanotubes and Ag doped ZnIn2S4 quantum dots for oxytetracycline detection. Talanta.

[4] Nam B., Ko T.-K., Hyun S.-K., Lee C. (2019). NO_2_ sensing properties of WO_3_-decorated In_2_O_3_ nanorods and In_2_O_3_-decorated WO_3_ nanorods. Nano Converg..

[5] Zhao F., Cao W., Wang P.-H., Wang J., Yu L., Qiao Z., Ding Z.-J. (2023). Fast and sensitive detection of CO by Bi-MOF-derived porous In_2_O_3_/Fe_2_O_3_ core-shell nanotubes. ACS Sens..

[6] Han C., Zhang X., Huang S., Hu Y., Yang Z., Li T.T., Li Q., Qian J. (2023). MOF-on-MOF-derived hollow Co_3_O_4_/In_2_O_3_ nanostructure for efficient photocatalytic CO_2_ reduction. Adv. Sci..

[7] Shi L., Benetti D., Wei Q., Rosei F. (2023). MOF-derived In_2_O_3_/CuO p-n heterojunction photoanode incorporating graphene nanoribbons for solar hydrogen generation. Small.

[8] Cao Y., Lu K., Chen Y., Zheng Q., Huang C., Jia N. (2023). In_2_O_3_/Bi_2_S_3_ S-scheme heterojunction-driven molecularly imprinted photoelectrochemical sensor for ultrasensitive detection of dlorfenicol. ACS Appl. Mater. Interfaces.

[9] Liu X., Zhang L., Li Y., Xu X., Du Y., Jiang Y., Lin K. (2021). A novel heterostructure coupling MOF-derived fluffy porous indium oxide with g-C_3_N_4_ for enhanced photocatalytic activity. Mater. Res. Bull..

[10] Ren J., Yuan K., Wu K., Zhou L., Zhang Y. (2019). A robust CdS/In_2_O_3_ hierarchical heterostructure derived from a metal–organic framework for efficient visible-light photocatalytic hydrogen production. Inorg. Chem. Front..

[11] Yang J., Zhu X., Yu Q., He M., Zhang W., Mo Z., Yuan J., She Y., Xu H., Li H. (2022). Multidimensional In_2_O_3_/In_2_S_3_ heterojunction with lattice distortion for CO_2_ photoconversion. Chinese J. Catal..

[12] Luo D., Peng L., Wang Y., Lu X., Yang C., Xu X., Huang Y., Ni Y. (2021). Highly efficient photocatalytic water splitting utilizing a WO_3_-x/ZnIn_2_S_4_ ultrathin nanosheet Z-scheme catalyst. J. Mater. Chem. A.

[13] Ding S., Medic I., Steinfeldt N., Dong T., Voelzer T., Haida S., Rabeah J., Hu J., Strunk J. (2023). Ultrathin defective nanosheet subunit ZnIn_2_S_4_ hollow nanoflowers for efficient photocatalytic hydrogen evolution. Small Struct..

[14] Lu P., Liu K., Liu Y., Ji Z., Wang X., Hui B., Zhu Y., Yang D., Jiang L. (2024). Heterostructure with tightly-bound interface between In_2_O_3_ hollow fiber and ZnIn_2_S_4_ nanosheet toward efficient visible light driven hydrogen evolution. Appl. Catal. B Environ..

[15] Wang J., Sun S., Zhou R., Li Y., He Z., Ding H., Chen D., Ao W. (2021). A review: Synthesis, modification and photocatalytic applications of ZnIn_2_S_4_. J. Mater. Sci. Technol..

[16] Lin Y., Fang W., Xv R., Fu L. (2022). TiO_2_ nanoparticles modified with ZnIn_2_S_4_ nanosheets and Co-Pi groups: Type II heterojunction and cocatalysts coexisted photoanode for efficient photoelectrochemical water splitting. Int. J. Hydrogen Energ..

[17] Liu M., Xiong J., Kong D., Liu Y., Wu H., Li F., Hu H., Wang D., Guo X., Jiao Y. (2025). Anchoring ZnIn_2_S_4_ nanosheets on oxygen-vacancy NiMoOx nanorods for efficient photocatalytic hydrogen evolution. Sep. Purif. Technol..

[18] Kong D., Hu X., Geng J., Zhao Y., Fan D., Lu Y., Geng W., Zhang D., Liu J., Li H. (2022). Growing ZnIn_2_S_4_ nanosheets on FeWO_4_ flowers with pn heterojunction structure for efficient photocatalytic H_2_ production. Appl. Surf. Sci..

[19] Wang M., Zhang G., Guan Z., Yang J., Li Q. (2021). Spatially separating redox centers and photothermal effect synergistically boosting the photocatalytic hydrogen evolution of ZnIn_2_S_4_ nanosheets. Small.

[20] Liu T., Shen H., Wang M., Feng Q., Chen L., Wang W., Zhang J. (2023). Fabrication of ZnIn2S4 nanosheets decorated hollow CdS nanostructure for efficient photocatalytic H_2_-evolution and antibiotic removal performance. Sep. Purif. Technol..

[21] Medetalibeyoglu H., Kotan G., Atar N., Yola M.L. (2020). A novel and ultrasensitive sandwich-type electrochemical immunosensor based on delaminated MXene@ AuNPs as signal amplification for prostate specific antigen (PSA) detection and immunosensor validation. Talanta.

[22] Zheng J., Zhao H., Ning G., Sun W., Wang L., Liang H., Xu H., He C., Zhao H., Li C. (2021). P A novel affinity peptide–antibody sandwich electrochemical biosensor for PSA based on the signal amplification of MnO_2_-functionalized covalent organic framework. Talanta.

[23] Samanta S., Bhattacharyya I.M., Prajapati A., Ron I., Shima-Edelstein R., Pikhay E., Greental D., Eisenberg-Lerner A., Rotfogel Z., Roizin Y. (2023). Specific and label-free sensing of prostate-specific antigen (PSA) from an ultrasmall drop of diluted human serum with the meta-nano-channel Silicon field-effect biosensor. Adv. Mate. Technol..

[24] Wang S., Guan B.Y., Lou X.W.D. (2018). Construction of ZnIn_2_S_4_-In_2_O_3_ hierarchical tubular heterostructures for efficient CO_2_ photoreduction. J. Am. Chem. Soc..

[25] Chang Y.-S., Choi M., Baek M., Hsieh P.-Y., Yong K., Hsu Y.-J. (2018). CdS/CdSe co-sensitized brookite H:TiO_2_ nanostructures: Charge carrier dynamics and photoelectrochemical hydrogen generation. Appl. Catal. B Environ..

