# Supplementary material for: Photoelectrochemical Biosensor Based on 1D In2O3 Tube Decorated with 2D ZnIn2S4 Nanosheets for Sensitive PSA Detection
Source: Nanomaterials (Basel). 2025 Jun 3;15(11):855. doi: 10.3390/nano15110855 (PMC12157784; doi:10.3390/nano15110855)
Supplement: Supplementary file 1 [file nanomaterials-15-00855-s001.zip › nanomaterials-3567405-supplementary.pdf]

Supplementary Materials

Photoelectrochemical Biosensor Based on 1D In_2O_3 Tube Decorated with 2D $ZnIn_2S_4$ Nanosheets for Sensitive PSA Detection

Huihui Shi [1,2], Jianjian Xu [3] and Yanhu Wang [2,*]

[1] Key Lab of MEMS of Ministry of Education, Southeast University, Nanjing 210096, China; shihuihui@seu.edu.cn

[2] Shandong Analysis and Test Center, Qilu University of Technology (Shandong Academy of Sciences), Jinan 250014, China

[3] Department of Food and Drug, Weihai Ocean Vocational College, Weihai 264300, China; jjjcs@whovc.edu.cn

* Correspondence: chm_wangyh@qlu.edu.cn

1. Results and Discussions

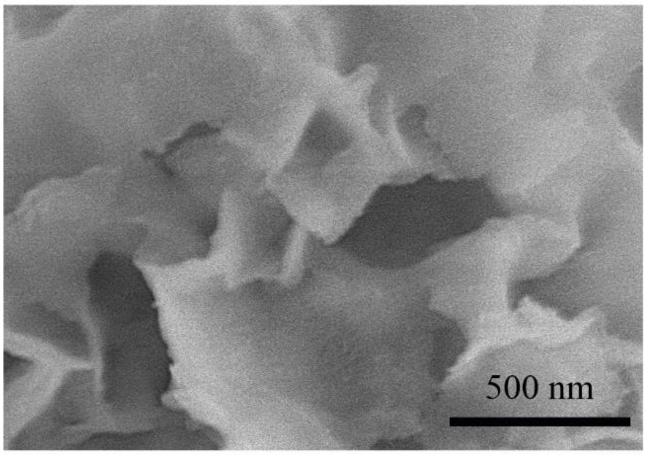

Figure S1. Enlarged SEM image of $ZnIn_2S_4$.

Table S1. Key data of EIS measurement in this study. In this model, electron-transfer resistance is the main factor, thus the solution resistance value is artificially zeroed to better demonstrate the change of electron-transfer resistance.

Substrate	R_{et} (Ω)	Warburg impedance
FTO	62.3	1.245E-005
FTO/In_2O_3-$ZnIn_2S_4$	252.4	1.999E-005
FTO/In_2O_3-$ZnIn_2S_4$/aptamer	633.3	2.589E-005
FTO/In_2O_3-$ZnIn_2S_4$/aptamer/BSA	1154.9	3.478E-005
